# A long-term gridded dataset of aboveground net primary productivity for global natural grasslands

**DOI:** 10.1038/s41597-026-06944-7

**Published:** 2026-02-27

**Authors:** Ziwei Chen, Dongsheng Zhao, Zhiyuan Zhang, Liming Zhang, Du Zheng

**Affiliations:** 1 University Key Lab of Soil Ecosystem Health and Regulation in Fujian, College of Resources and Environment, Fujian Agriculture and Forestry University, Fuzhou, 350002 China; 2 Key Laboratory of Land Surface Pattern and Simulation, Institute of Geographic Sciences and Natural Resources Research, Chinese Academy of Sciences, Beijing, 100101 China

**Keywords:** Grassland ecology, Carbon cycle, Agroecology

## Abstract

A long-term dataset of aboveground net primary productivity (ANPP) for global natural grasslands is essential for carbon dynamics modeling and sustainable land management. However, existing datasets are limited: they often fail to separate above- and below-ground productivity or reflect only post-disturbance conditions. To address these gaps, we developed a gridded annual ANPP dataset using machine learning, spanning historical (1958–2023) and future (2015–2100) periods. Historical ANPP data were derived from TerraClimate at 1/24° spatial resolution, while future projections came from CMIP6 models under SSP245 and SSP585 scenarios at 1/2° resolution. Our model performed robustly (R^2^ = 0.675 ± 0.009), showing temporal and spatial reliability through cross-validation with published products. Notably, systematic ANPP underestimation occurs in high-productivity regions (>700 g m^−2^) due to sparse field observations, so values in these areas should be interpreted with caution. Our dataset provides a spatially explicit baseline of climate-driven productivity, supporting precise evaluation of human impacts on grasslands and informing adaptive management under climate change.

## Background & Summary

Grasslands cover around half of the world’s ice-free land^1^ and provide about one-third of terrestrial net primary productivity (NPP)^2^. Aboveground NPP (ANPP) directly supplies energy for animal products and underpins livestock farming^3^. ANPP also reflects the plant carbon uptake, affecting the global carbon balance^4^. Mapping the global distribution of grassland ANPP is essential for assessing the sustainability of grazing ecosystems and advancing carbon cycle research.

Climate change and human activities, especially livestock grazing, significantly influence grassland ANPP^5,6^. Quantifying the isolated impacts of human activities on ANPP facilitates the development of targeted grassland management and restoration strategies^7^, which requires knowledge of baseline levels of ANPP in natural grasslands without human disturbance^8^. Additionally, since climate change is a long-term issue^9^, compiling multidecadal ANPP datasets for natural grasslands is indispensable for refining ecological models and informing policymaking.

However, the estimation of ANPP in natural grasslands encounters methodological challenges. Climate-based NPP estimations, such as the Miami model^10^, and satellite-based NPP estimations, such as the light use efficiency model^11^, often neglect the NPP allocation within plants, biasing ANPP estimates. Moreover, satellite observations primarily reflect anthropogenically modified vegetation conditions, rendering them unsuitable for reconstructing pre-disturbance natural grassland ANPP^12^. While ecosystem process models can simulate carbon uptake and allocation in natural grasslands^13^, they rely on extensive plant physiological parameters^14^, posing significant constraints on global-scale calibration and validation^15^.

Accumulated field observations of grassland ANPP and developed machine learning techniques have fostered global simulation of natural grassland ANPP. For instance, Sun, Feng *et al*.^8^ used the random forest model to generate a global gridded dataset of natural grassland ANPP, based on site-specific observations. However, their study has some limitations. First, it provides only multi-year averages without time-varying data. Second, mismatches in timing between ANPP observations and covariates reduce prediction accuracy. Lastly, the large number of covariates and the lack of access to some of them limit model generalization and data reuse.

**Fig. 1:**
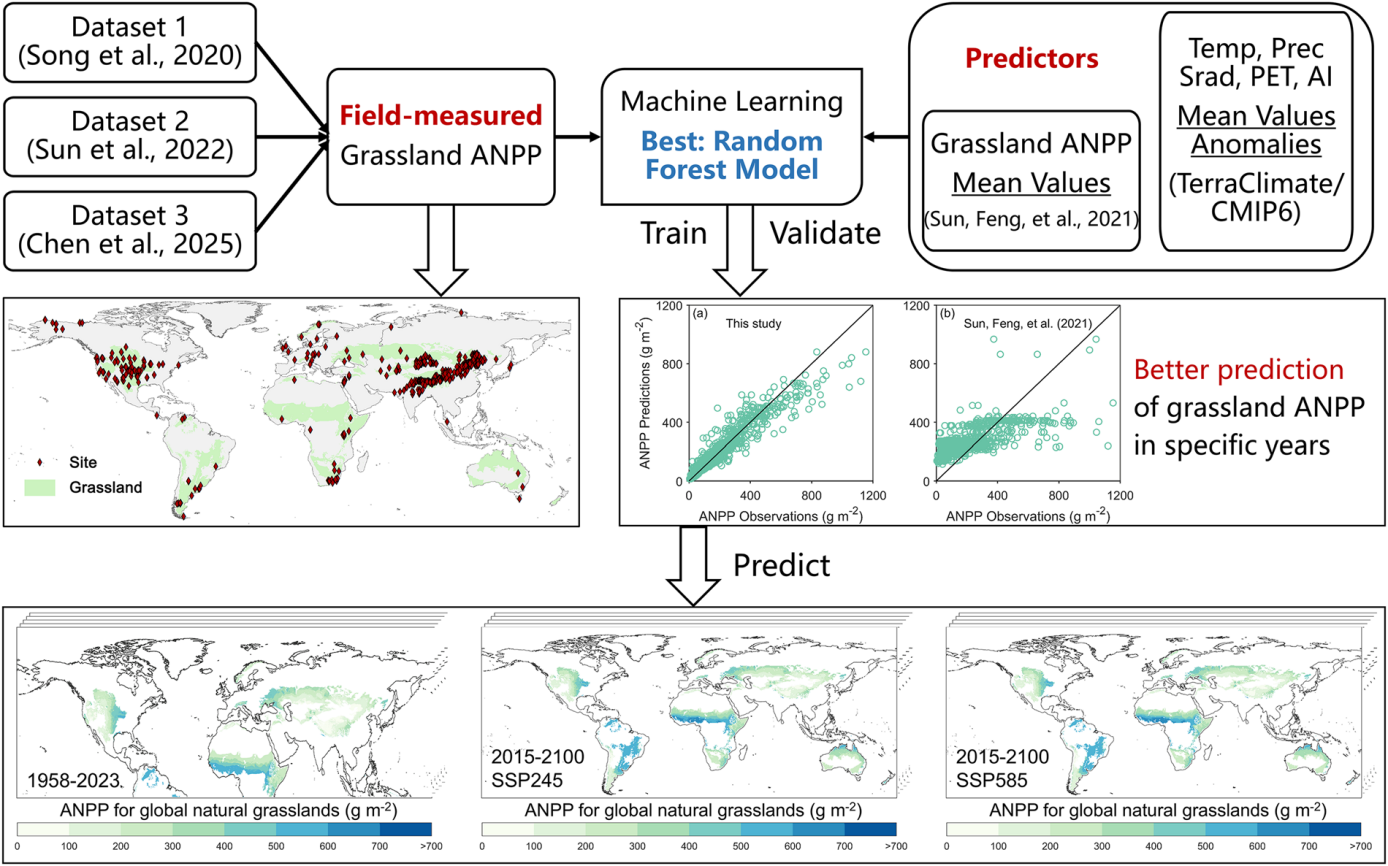
A schematic overview of the study and experimental design.

To address these gaps, we developed a new framework to simulate annual ANPP over the long term in natural grasslands (Fig. 1). By combining multi-year ANPP averages from Sun, Feng *et al*.^8^ with annual weather records, we effectively captured the interannual dynamics of ANPP while minimizing the complexity of model inputs. Ultimately, we established a gridded annual ANPP dataset for global natural grasslands^16^, consisting of two subsets: one based on the TerraClimate database, spanning 1958–2023 at 1/24° resolution; the other derived from CMIP6 projections under SSP245 and SSP585 climate scenarios, from 2015 to 2100 at 1/2° resolution. The dataset allows researchers to investigate how global grassland ANPP responds to climate change and human activities while also supporting precise assessments of grassland carrying capacity.

## Methods

This study developed a spatially explicit gridded dataset of annual grassland ANPP by integrating published multi-year mean ANPP data with field observations and time-varying meteorological records. This study operates under the premise that interannual ANPP variability is primarily attributed to climatic factors rather than time-invariant regulators, e.g., soil and topography.

The main steps of this study were as follows:

1. Field observations of natural grassland ANPP from undisturbed sites were collected from multiple sources and compiled into a comprehensive dataset.
2. Multi-year mean ANPP values from the published dataset were spatially matched and extracted to align with field-measured ANPP locations.
3. Historical and future meteorological data were aggregated into annual values.
4. Annual anomalies of each climate variable relative to their 1970–2000 multi-year means were quantified.
5. Six machine learning models were used to model grassland ANPP, with the top-performing one selected based on predictive accuracy.
6. The hyperparameters of the top-performing model were optimized via grid search, and the model was trained across optimal hyperparameters.
7. The best model was applied to historical and future climate scenarios to generate gridded ANPP estimates.
8. Dataset reliability was validated by benchmarking against published ANPP datasets.

## Data

### Field-measured grassland ANPP data

**Fig. 2:**
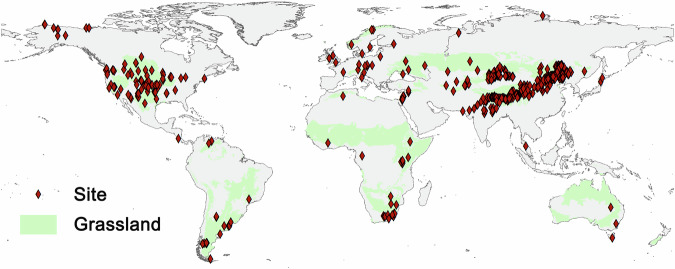
Spatial distribution of aboveground net primary productivity (ANPP) measurement sites. Global grassland distribution was derived from Dixon *et al*.^57^.

We integrated three distinct datasets into the comprehensive ANPP field-measured dataset for natural grasslands to date. The data sources included: (1) the global change experiment dataset by Song *et al*.^17^ (10.6084/m9.figshare.7442915.v13); (2) the grassland ANPP field observation dataset by Sun *et al*.^18^ (10.5061/dryad.7sqv9s4vv); and (3) the livestock grazing experiment dataset extracted from Chen *et al*.^19^. Records were retained if two criteria were met: (1) explicit reporting of sampling location and year; and (2) derivation from natural grasslands (control sites without grazing). After deduplication, the final dataset comprises 1,503 unique records—100 from Song *et al*.^17^, 1,284 from Sun *et al*.^18^, and 119 from Chen *et al*.^19^. Observations in this dataset are spatially extensive, biome-diverse, and globally representative, spanning all continents except Antarctica (Fig. 2).

### Mean annual grassland ANPP gridded data

We sourced the global gridded ANPP dataset for natural grasslands from Sun, Feng *et al*.^8^ (https://zenodo.org/records/5554579), which features a 1-km spatial resolution and reflects the multi-year average spanning 1970–2000. Subsequently, we spatially aligned and extracted mean annual ANPP values for sites in the field measurement dataset based on their geographic locations.

### Time-varying climate data

We obtained time-varying data for five climate variables—temperature, precipitation, solar radiation, potential evapotranspiration, and aridity index since Sun, Feng *et al*.^8^ incorporated these variables in their grassland ANPP simulation. Potential evapotranspiration was calculated via the Penman-Monteith method^20^, while the aridity index was defined as the ratio of precipitation to potential evapotranspiration.

Historical climate data were sourced from the TerraClimate database^21^ (10.7923/G43J3B0R), which was developed based on the WorldClim v1.4^22^, Climate Research Unit time series (CRU TS) v4.0^23^, and the Japanese 55-year Reanalysis (JRA-55) datasets^24^. This database provides monthly climate data at 1/24° resolution for 1958–2023, later aggregated to annual values.

We obtained future climate data from the Coupled Model Intercomparison Project Phase 6 (CMIP6, https://esgf-node.llnl.gov/search/cmip6), including monthly climate simulations from 13 CMIP6 models (Table 1) and potential evapotranspiration derived from these simulations^25^ (10.5281/zenodo.7789759). All CMIP6 data were bias-corrected using the Quantile Delta Mapping (QDM) method^26^ to align with the TerraClimate baseline (1970–2000). This approach preserves the long-term trends of all quantiles of the climate distribution, thereby improving the reliability of our future projections. We evaluated two Shared Socioeconomic Pathways (SSPs) scenarios: SSP245 (low-emission) and SSP585 (high-emission). All CMIP6 data were spatially resampled to 1/2° resolution using bilinear interpolation^27^ and aggregated to annual values.

**Table 1:** Basic information of the 13 CMIP6 models used in this study.

Model	Institute	Country	Resolution
ACCESS-CM2	Commonwealth Scientific and Industrial Research Organisation	Australia	1.875° × 1.25°
ACCESS-ESM1-5	Commonwealth Scientific and Industrial Research Organisation	Australia	1.875° × 1.2414°
CMCC-CM2-SR5	Fondazione Centro Euro-Mediterraneo	Italy	1.25° × 0.9375°
CMCC-ESM2	Fondazione Centro Euro-Mediterraneo	Italy	1.25° × 0.9375°
EC-Earth3	Swedish Meteorological and Hydrological Institute *et al*.	Europe	0.7031° × 0.7031°
GFDL-ESM4	National Oceanic and Atmospheric Administration, Geophysical Fluid Dynamics Laboratory	America	1.25° × 1.0°
INM-CM4-8	Institute for Numerical Mathematics	Russia	2.0° × 1.5°
INM-CM5-0	Institute for Numerical Mathematics	Russia	2.0° × 1.5°
IPSL-CM6A-LR	Institut Pierre Simon Laplace	France	2.5° × 1.2587°
MIROC6	Atmosphere and Ocean Research Institute, The University of Tokyo, Japan Agency for Marine-Earth Science and Technology	Japan	1.4063° × 1.4063°
MPI-ESM1-2-HR	Max Planck Institute for Meteorology	Germany	0.9375° × 0.9375°
MPI-ESM1-2-LR	Max Planck Institute for Meteorology	Germany	1.875° × 1.9565°
MRI-ESM2-0	Meteorological Research Institute	Japan	1.25° × 1.25°

### Satellite-derived NPP data

We validated our ANPP dataset using two NPP products: GLASS (Global LAnd Surface Satellite) NPP and BEPS (Boreal Ecosystem Productivity Simulator) NPP. The GLASS NPP relies on vegetation indices derived from the AVHRR (Advanced Very High Resolution Radiometer) to quantify vegetation growth^28^. It employs the revised EC-LUE (Eddy Covariance-Light Use Efficiency) model to simulate gross primary productivity (GPP) and integrates the ratio of autotrophic respiration to GPP simulated by 10 dynamic global vegetation models from the TRENDY project to derive NPP^28^. This dataset (https://www.glass.hku.hk/archive/NPP/AVHRR/0.05D) spans 1982–2018 with a 0.05° spatial resolution.

The BEPS model is a process-based diagnostic model that fuses AVHRR and MODIS (Moderate Resolution Imaging Spectroradiometer) data to simulate vegetation carbon input^29^. It calculates GPP for sunlit and shaded leaves using the Farquhar model, accounting for growth and maintenance respiration fluxes to derive NPP^29^. The BEPS NPP dataset (10.12199/nesdc.ecodb.2016YFA0600200.02.002) covers 1981–2019 at a 0.0727° spatial resolution.

### Process-based NPP data

We validated the temporal reliability of our ANPP dataset with independent NPP outputs from 20 DGVMs in TRENDY v14 (Table 2). These models simulate vegetation carbon dynamics through distinct ecological mechanisms, providing true independence from our machine-learning approach^30^. We extracted monthly NPP outputs under the S2 scenario (historical climate change with constant pre-industrial land use) for 1958–2023 (https://mdosullivan.github.io/GCB). This scenario is widely adopted to isolate climate-driven signals in vegetation productivity^31^. All DGVM outputs were to 1/2° by bilinear interpolation, aggregated to annual values, and ensemble-averaged.

**Table 2:** Basic information of the 20 dynamic global vegetation models used in this study.

Model	Institute	Country	Resolution
CABLE-POP	University of Technology Sydney	Australia	1° × 1°
CLASSIC	Environment and Climate Change Canada	Canada	1° × 1°
CLM-FATES	Lawrence Berkeley National Laboratory	America	1.8947° × 2.5°
CLM6.0	National Center for Atmospheric Research	America	0.9424° × 1.25°
DLEM	Boston College	America	0.5° × 0.5°
E3SM	Lawrence Livermore National Laboratory	America	0.9424° × 1.25°
EDv3	University of Maryland	America	0.5° × 0.5°
ELM-FATES	Lawrence Berkeley National Laboratory	America	1.8947° × 2.5°
GDSTEM	University of California, Davis	America	0.5° × 0.5°
IBIS	University of Wisconsin–Madison	America	0.5° × 0.5°
ISAM	University of Illinois at Urbana-Champaign	America	0.5° × 0.5°
JSBACH	Max Planck Institute for Meteorology	Germany	1.8606° × 1.875°
JULES-ES	Centre for Ecology & Hydrology	United Kingdom	0.5° × 0.5°
LPJ-EOSIM	Potsdam Institute for Climate Impact Research	Germany	0.5° × 0.5°
LPJ-GUESS	Karlsruhe Institute of Technology	Germany	0.5° × 0.5°
LPX-Bern	University of Bern	Switzerland	0.5° × 0.5°
ORCHIDEE	Institute Pierre Simon Laplace	France	0.5° × 0.5°
SDGVM	Oak Ridge National Laboratory	America	1° × 1°
VISIT-UT	The University of Tokyo	Japan	0.5° × 0.5°
iMAPLE	Nanjing University of Information Science & Technology	China	1° × 1°

### Soil and topography data

To evaluate the contribution of time-invariant regulators, we compiled soil and topography variables for all field sites. Soil data were extracted from the WISE30sec database^32^ (https://data.isric.org/geonetwork/srv/api/records/dc7b283a-8f19-45e1-aaed-e9bd515119bc), which provides global soil property estimates at 30 arc-second resolutions. Selected variables included soil pH, total nitrogen content (g kg^−1^), soil organic carbon content (g kg^−1^), soil carbon-to-nitrogen (C/N) ratio, clay content (%), silt content (%), and bulk density (kg m^−3^). Topographic data were derived from the Shuttle Radar Topography Mission (SRTM) digital elevation model (DEM) at 30 arc-second resolutions^33^ (https://worldclim.org/data/worldclim21.html). Elevation (m), slope (°), and aspect (°) were extracted for each site. These variables were spatially matched to measurement sites and incorporated into an extended model to test whether explicitly representing static regulators improves predictive performance compared to the baseline model, which implicitly captures these effects through multi-year mean ANPP.

## Methodology

### Climatic anomalies calculation

Annual grassland ANPP was predicted using machine learning models, with predictors including sampling-year climate anomalies. The climate anomalies accounted for deviations between the ANPP baseline and actual measurements. Due to strong correlations between sampling year and mean annual climate data, climate anomalies were used as model inputs rather than raw data to mitigate the impact of multicollinearity.

For the four climate factors except temperature (precipitation, solar radiation, potential evapotranspiration, and aridity index), anomalies were calculated as:

1 $${X}_{{ano}}=\frac{{X}_{{act}}-{X}_{{mean}}}{{X}_{{mean}}}$$

where ${X}_{{ano}}$ was the climate anomaly, ${X}_{{act}}$ was the actual climate data observed in the sampling year, and ${X}_{{mean}}$ was the mean annual climate data (1970–2000).

Since the mean annual air temperature can approach 0 °C in some sites, calculating temperature anomaly using Eq. (1) may produce extreme outliers and distort the direction of changes, thereby masking real ecological signals. Therefore, the temperature anomaly was calculated as^34^:

2 $${X}_{{ano}}={X}_{{act}}-{X}_{{mean}}$$

### Model comparison and selection

Machine learning techniques are particularly advantageous for modeling complex, highly nonlinear ecosystems^35^, as they do not rely on the reliance on simplistic statistical assumptions or rigidly prescribed variable interactions that constrain traditional statistical methods^36^. We evaluated six algorithms to simulate grassland ANPP: linear model, support vector machine, random forest, artificial neural network, bagged classification and regression tree (CART), and eXtreme Gradient Boosting (XGBoost). Comprehensive descriptions of these algorithms can be found in references^37–39^.

Our models used measured ANPP as the dependent variable, with mean annual ANPP, climatic baselines, and annual climatic anomalies as predictors. We employed cross-validation to evaluate model performance and prevent overfitting. To comprehensively assess generalization ability and evaluate potential overestimation due to spatial autocorrelation, we systematically compared three cross-validation strategies^40^. First, random 10-fold cross-validation served as the baseline. Second, spatial-block cross-validation divided the data into geographically contiguous blocks assigned to different folds. This ensured spatial independence between training and validation sets, reducing inflated performance estimates caused by spatial autocorrelation. Third, environmentally-stratified cross-validation clustered observations into 10 climate classes using k-means^41^ on multi-year mean ANPP and five climatic variables. Each class was then distributed proportionally across folds to preserve environmental representativeness and avoid extrapolation bias. Each strategy was repeated 10 times with different random seeds. Model performance was measured via the coefficient of determination (R^2^), root mean square error (RMSE), and mean absolute error (MAE). Differences between validation strategies were assessed with repeated-measures ANOVA and Tukey’s HSD test.

**Fig. 3:**
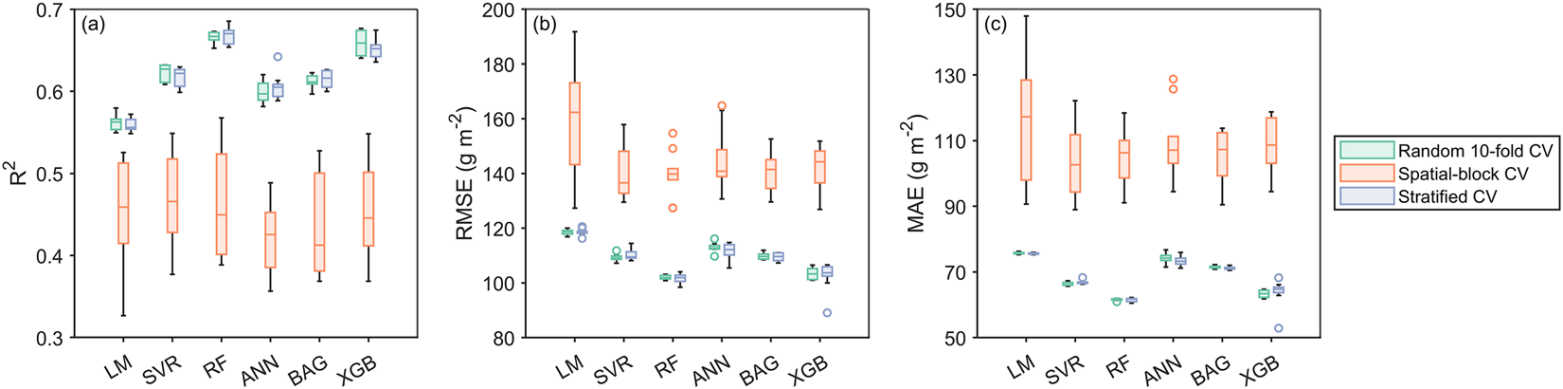
Performance comparison of six machine learning algorithms under three cross-validation (CV) strategies. (**a**) Coefficient of determination (R^2^), (**b**) root mean square error (RMSE), and (**c**) mean absolute error (MAE). Higher R^2^ values and lower RMSE/MAE values indicate superior predictive performance. For each algorithm, three validation strategies are displayed from left to right: random 10-fold CV, spatial-block CV, and environmentally-stratified CV. Box plots show medians, quartiles, non-outlier ranges, and outliers from 10 repetitions with different random seeds. LM = linear model, SVR = support vector machine, RF = random forest, ANN = artificial neural network, BAG = bagged classification and regression tree, and XGB = eXtreme Gradient Boosting.

Under both random 10-fold and environmentally-stratified cross-validation, the random forest model consistently performed best, followed by the XGBoost model (*p* < 0.001, Fig. 3). This consistency across strategies reinforced confidence in the random forest model’s superiority. Spatial-block cross-validation reduced performance and increased variability for all models (Fig. 3). This occurs because the strategy disrupts continuous spatial environmental gradients. As a result, large differences arise in predictive space between training and validation sets, yielding overly pessimistic error estimates^40^. Given that our goal is to produce a global dataset covering the full environmental gradient, not extreme extrapolation, environmentally- stratified cross-validation offers a more balanced evaluation framework. We therefore tuned and trained the random forest model exclusively within this framework.

**Fig. 4:**
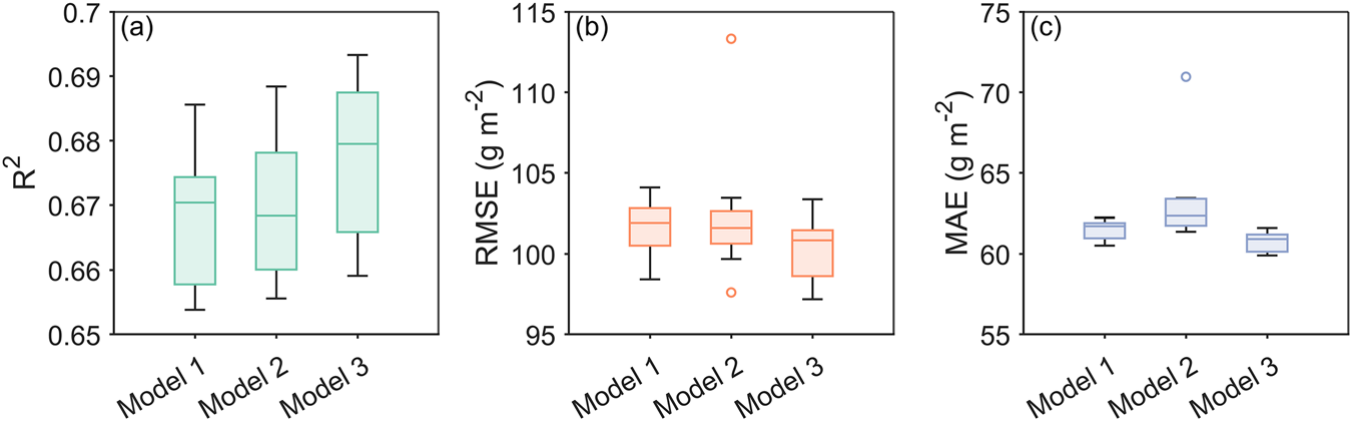
Performance comparison of three model setups under environmentally-stratified cross-validation. (**a**) Coefficient of determination (R^2^), (**b**) root mean square error (RMSE), and (**c**) mean absolute error (MAE). Higher R^2^ values and lower RMSE/MAE values indicate superior predictive performance. Model 1 is the baseline random forest model incorporating multi-year mean ANPP, climatic baselines, and anomalies. Model 2 represents the ensemble mean of random forest and eXtreme Gradient Boosting predictions. Model 3 extends the random forest model with soil and topographic factors. Box plots show medians, quartiles, non-outlier range, and outliers across 10 repetitions with different random seeds.

This study assumes that interannual variability in grassland ANPP is mainly controlled by climatic factors, while time-invariant regulators (e.g., soil and topography) are largely reflected in multi-year mean ANPP. To validate this, we tested whether including static environmental factors could improve model performance. An extended model was built by incorporating ten soil and topographic predictors (see *Soil and topography data*), and its performance was compared to the baseline model using environmentally-stratified cross-validation. Results showed statistically significant yet marginal improvement with the extended model (*p* < 0.001, Fig. 4).

Given that random forest performed best, we further examined whether combining it with other high-performing models could reduce uncertainty. We created an ensemble model by averaging predictions from random forest and XGBoost (the second-best) models. However, this ensemble did not significantly outperform the single random forest model (*p* > 0.05, Fig. 4). Since neither extended variables nor model integration yielded substantial gains, we retained the original random forest model for its parsimony, computational efficiency, and reproducibility.

### Model tuning and prediction

Random forest model tuning targeted two hyperparameters^42^: the number of trees (ntree) and the number of random variables at each node (mtry). We evaluated 25 parameter combinations (ntree: 200, 400, 600, 800, 1000; mtry: 2, 4, 6, 8, 10) over 10 repeated training cycles. In each cycle, model performance was assessed via environmentally-stratified cross-validation using field-measured grassland ANPP, with R^2^ guiding hyperparameter selection. The optimal combination was ntree = 400 and mtry = 2, achieving a cross-validated R^2^ value of 0.672 (Table 3).

**Table 3:** Hyperparameter selection for the grassland ANPP prediction model.

mtry	ntree	Coefficient of determination (R^2^)										Means
		1	2	3	4	5	6	7	8	9	10	
2	200	0.672	0.669	0.671	0.672	0.672	0.669	0.672	0.670	0.671	0.671	0.6709
4	200	0.669	0.670	0.672	0.671	0.671	0.673	0.670	0.672	0.672	0.673	0.6713
6	200	0.672	0.673	0.674	0.672	0.667	0.670	0.671	0.672	0.670	0.670	0.6712
8	200	0.669	0.673	0.669	0.669	0.673	0.670	0.669	0.668	0.671	0.671	0.6702
10	200	0.671	0.668	0.671	0.671	0.669	0.669	0.670	0.671	0.669	0.669	0.6697
***2***	***400***	***0.672***	***0.672***	***0.672***	***0.672***	***0.673***	***0.673***	***0.673***	***0.671***	***0.673***	***0.669***	***0.6720***
4	400	0.668	0.673	0.673	0.671	0.670	0.671	0.671	0.669	0.670	0.672	0.6707
6	400	0.671	0.670	0.670	0.674	0.671	0.669	0.671	0.672	0.673	0.671	0.6712
8	400	0.672	0.671	0.669	0.669	0.669	0.669	0.672	0.669	0.672	0.671	0.6704
10	400	0.671	0.672	0.672	0.670	0.669	0.669	0.671	0.671	0.670	0.670	0.6705
2	600	0.671	0.671	0.671	0.672	0.675	0.673	0.670	0.669	0.672	0.672	0.6717
4	600	0.671	0.671	0.671	0.671	0.671	0.672	0.673	0.672	0.673	0.673	0.6719
6	600	0.669	0.670	0.671	0.671	0.671	0.671	0.670	0.669	0.672	0.672	0.6706
8	600	0.670	0.669	0.670	0.672	0.672	0.671	0.671	0.672	0.671	0.672	0.6709
10	600	0.671	0.670	0.669	0.673	0.670	0.669	0.671	0.669	0.668	0.671	0.6702
2	800	0.671	0.675	0.670	0.670	0.671	0.673	0.671	0.670	0.670	0.672	0.6711
4	800	0.673	0.672	0.671	0.671	0.673	0.672	0.670	0.669	0.672	0.672	0.6715
6	800	0.671	0.670	0.672	0.672	0.672	0.668	0.670	0.669	0.671	0.672	0.6708
8	800	0.670	0.669	0.668	0.673	0.669	0.673	0.670	0.671	0.671	0.670	0.6703
10	800	0.669	0.669	0.670	0.669	0.669	0.668	0.670	0.670	0.671	0.669	0.6694
2	1000	0.672	0.670	0.671	0.670	0.671	0.672	0.673	0.671	0.673	0.670	0.6712
4	1000	0.672	0.672	0.673	0.671	0.672	0.669	0.673	0.673	0.670	0.672	0.6718
6	1000	0.671	0.669	0.672	0.669	0.672	0.671	0.671	0.670	0.671	0.672	0.6707
8	1000	0.672	0.671	0.670	0.671	0.671	0.668	0.672	0.669	0.670	0.670	0.6702
10	1000	0.670	0.673	0.671	0.670	0.669	0.668	0.672	0.670	0.670	0.670	0.6704

Using the optimal hyperparameters, we trained random forest models 500 times to generate 500 independent models. Each model underwent environmentally-stratified cross-validation for performance assessment. Ideally, ensemble predictions from all 500 models would provide robust grassland ANPP estimates and uncertainty quantification^43^. However, given the computational and temporal constraints of high-resolution, long-term ANPP predictions, we selected the single best-performing model for final predictions, rather than averaging all 500.

Annual grassland ANPP was estimated at 1/24° spatial resolution for 1958–2023 by integrating mean annual ANPP data with time-varying climate variables from the TerraClimate database. Additionally, natural grassland ANPP projections for 2015–2100 at 1/2° resolution were derived from SSP245 and SSP585 scenario data from 13 CMIP6 models.

### Spatial validation

Multiple global ANPP datasets have been developed using statistical^44–46^ and machine learning methods^8^. To validate our ANPP dataset’s spatial distribution, comparisons were performed with these four published datasets. Notably, most rely on 1970–2000 climatic averages, so our corresponding results during this period were used for comparisons. For Del Grosso *et al*.^45^, which uses 1961–1990 climate averages, we substituted 1970–2000 climatic averages into their statistical model to generate comparable ANPP estimates.

Specifically, we conducted three quantitative spatial analyses to quantify the spatial pattern consistency between this dataset and existing products. First, we classified continuous ANPP data into five categories based on their cumulative distribution percentiles (0, 20, 40, 60, 80, 100%) and then calculated Kappa statistics between our dataset and published datasets^47^. Kappa values above 0.6 indicate substantial agreement^48^. Second, we plotted a Taylor diagram to visualize comparisons between published datasets and our dataset^49^. This diagram simultaneously displays three key statistics: correlation coefficients with our dataset, standard deviations for each dataset, and centered root mean square errors. This allows for an intuitive assessment of each dataset’s similarity to our dataset in terms of spatial variability magnitude and pattern. Third, we computed global and local Moran’s I, and generated local indicators of spatial association (LISA) cluster maps to evaluate whether our dataset exhibits realistic spatial autocorrelation patterns comparable to prior studies^50^.

### Temporal validation

Direct validation of our dataset’s temporal reliability (i.e., the accuracy of interannual variability) is hindered by the absence of a long-term global grassland ANPP dataset suitable for benchmarking. The inherent limitations of satellite-based NPP products also compromise their utility for indirect validation. First, ANPP constitutes the aboveground portion of NPP, and its proportion relative to total NPP varies with biome types and environmental conditions^51^. Empirical conversions from NPP to ANPP inherently introduce uncertainties. Second, satellite-derived NPP products often lack sufficient temporal coverage. For example, MOD17, one of the most widely used remote sensing NPP products, is only available post-2000^52^, precluding comparisons with the pre-2000 portion of our dataset. Third, these products rely on vegetation indices (e.g., NDVI, SIF) and thus reflect real-world conditions, failing to isolate anthropogenic disturbances like grazing.

Despite these constraints, we preliminarily validated the temporal reliability of our dataset through three approaches. First, we assessed systematic model bias by analyzing the relationship between model residuals (differences between observed and predicted ANPP) and sampling years. Since residuals theoretically follow a normal distribution, unusually large residuals may correlate with large ANPP values or sample sizes. To test whether residual outliers could be attributed to observed data characteristics, we grouped sampling years into five-year intervals and calculated the relative residual standard deviation (standard deviation of residuals divided by mean observed ANPP) and sample size for each group. Second, we compared our dataset with two long-term NPP products—GLASS NPP (1982–2018) and BEPS NPP (1981–2019)—under the assumptions of a stable “ANPP/NPP ratio” and “anthropogenic disturbance intensity” within individual grids. It has been indicated that ANPP/NPP ratios are primarily regulated by mean annual precipitation^46^. For a specific grid cell, mean annual precipitation is a constant, so the ANPP/NPP ratio is also considered temporally stable. We computed correlations between remote sensing NPP and our predicted ANPP for each grid to evaluate their dynamic consistency. Globally positive correlations would reinforce confidence in our dataset’s temporal accuracy. Third, to overcome temporal coverage limitations of satellite products and provide a more independent assessment, we validated our dataset using 20 DGVMs from the TRENDY project. For each grid cell, we calculated the correlation between the ANPP time series and the DGVMs’ NPP outputs (1958–2023). We assessed the robustness of our ANPP dataset’s temporal dynamics based on the 20 DGVMs and the TRENDY ensemble mean.

## Data Records

The global gridded dataset of natural grassland ANPP developed in this study is publicly accessible at 10.5281/zenodo.18171957^16^. The dataset comprises two compressed files: “historical_ANPP_map” and “future_ANPP_map”. After decompression, all files adopt the GeoTIFF format with annual temporal resolution. ANPP data are measured in g m^−2^, and NoData values are set to NaN.

The “historical_ANPP_map” dataset, derived from TerraClimate data, features a 1/24° spatial resolution. It follows the naming convention map_YYYY.tif, where YYYY corresponds to the years 1958–2023.

The “future_ANPP_map” dataset, derived from CMIP6 data, features a 1/2° resolution. It follows the naming convention map_MODEL_SSP_YYYY.tif, where MODEL represents the 13 CMIP6 models (Table 1), SSP denotes the SSP245 and SSP585 scenarios, and YYYY corresponds to the years 2015–2100. To enable users to assess uncertainties in future projections, we provide statistical layers for each year based on the ensemble of 13 models. They follow the naming convention map_STATISTIC_SSP_YYYY.tif, where STATISTIC denotes the mean, standard deviation (std), median, 5th percentile (p05), and 95th percentile (p95).

## Technical Validation

### Model validation based on field measurements

**Fig. 5:**
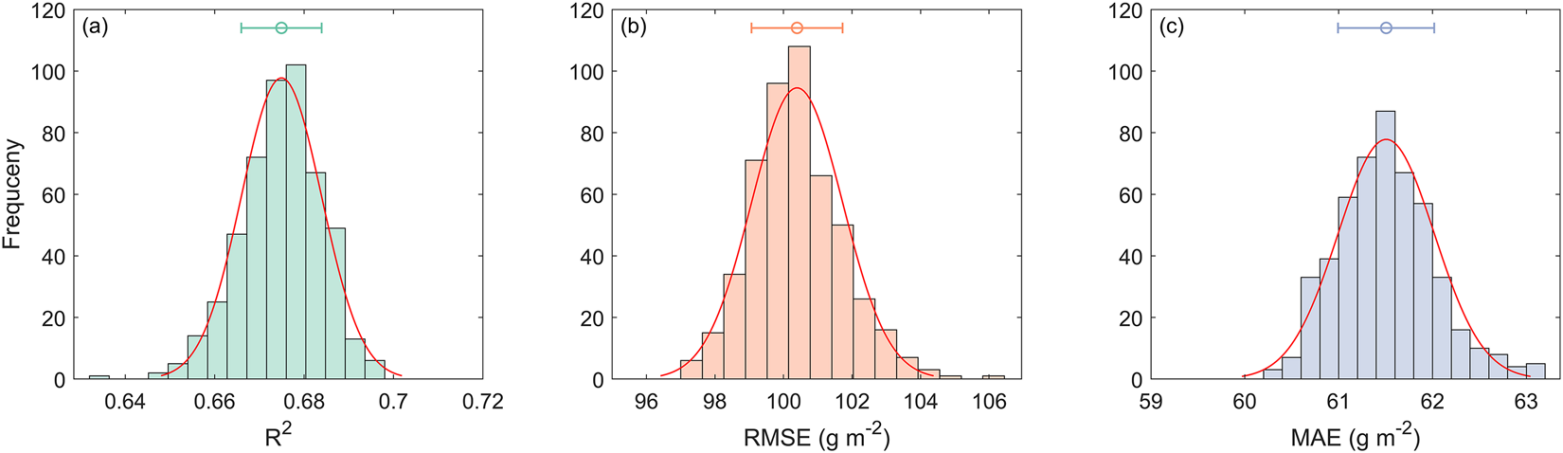
Performance distributions across 500 random forest models. (**a**) R^2^ (coefficient of determination), (**b**) RMSE (root mean square error), and (**c**) MAE (mean absolute error). Red curves denote fitted normal distributions, scatters indicate mean values, and error bars represent standard deviations.

Based on the optimized hyperparameters (ntree = 400 and mtry = 2), 500 random forest models were generated by training on the complete dataset. The performance of these models exhibited normal distribution characteristics (Fig. 5). Specifically, R^2^ was 0.675 ± 0.009, RMSE was 100.4 ± 1.3 g m^−2^, and MAE was 61.5 ± 0.5 g m^−2^ (mean ± standard deviation). These results demonstrate the robust predictive performance of random forest models.

**Fig. 6:**
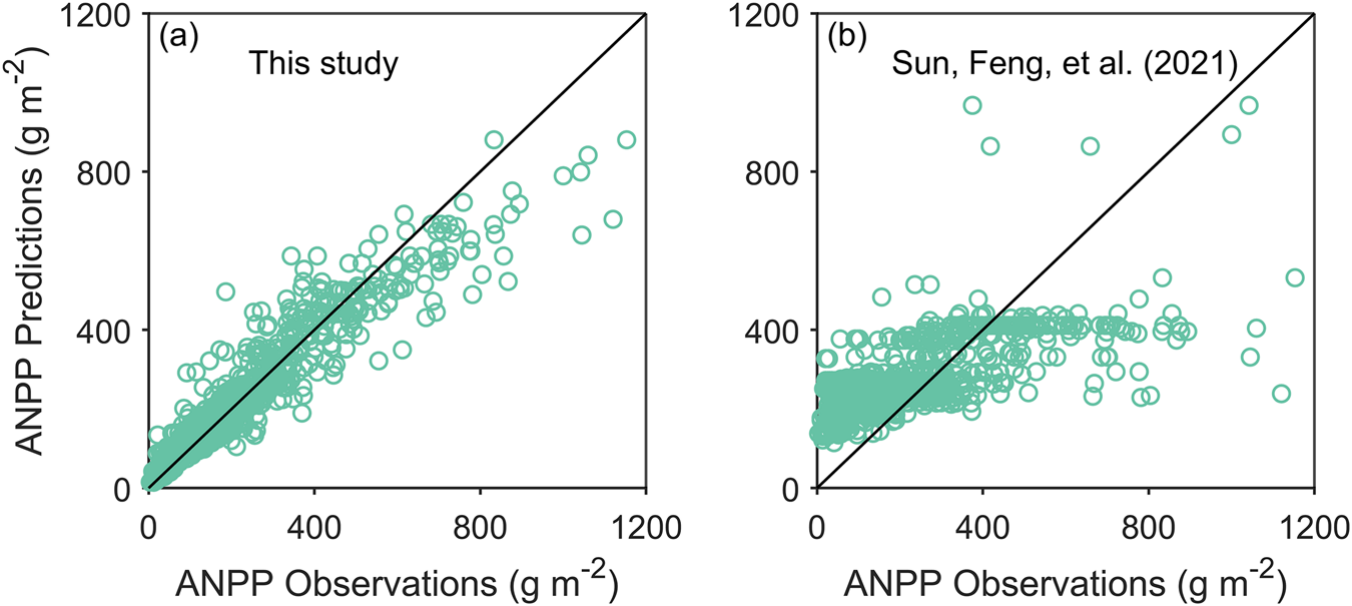
Comparison of observed and predicted aboveground net primary productivity (ANPP) for natural grasslands. (**a**) Specific-year results from this study and (**b**) multi-year average results from Sun, Feng *et al*.^8^.

Given computational and time constraints, we employed the top-performing model from the 500 candidates for grassland ANPP prediction. The resulting ANPP dataset demonstrates overall reliability as simulated and measured values were uniformly distributed around the 1:1 line (Fig. 6a). Further validation against Sun, Feng *et al*.^8^ confirmed the model’s superior accuracy in specific-year estimates (Fig. 6), attributed to its incorporation of interannual climate variability.

### Spatial reliability assessment

**Fig. 7:**
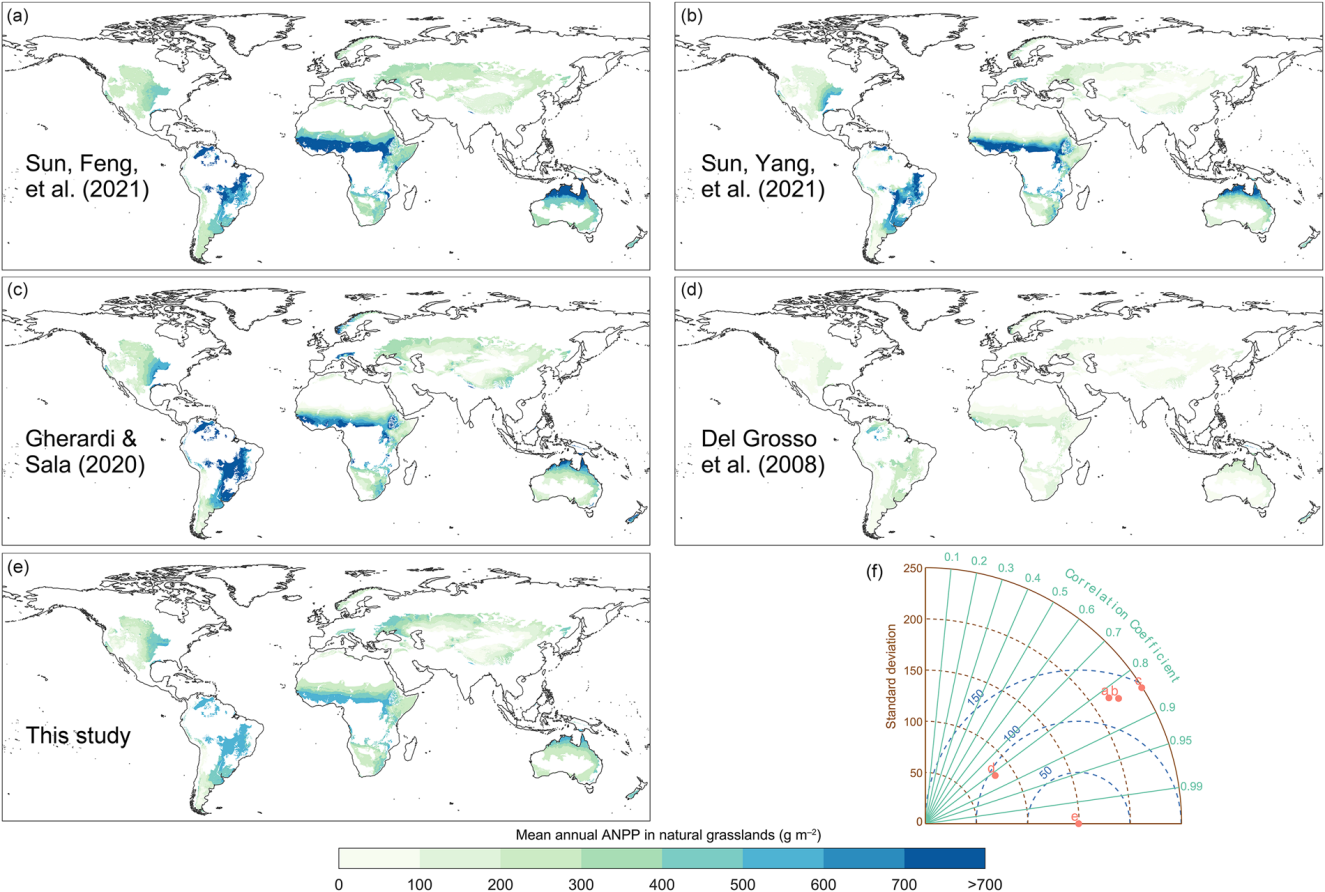
Spatial distribution and quantitative comparison of global grassland aboveground net primary productivity (ANPP) datasets. (**a**–**d**) Multi-year mean ANPP (1970–2000) from four published datasets, (**e**) multi-year mean ANPP (1970–2000) from this study, and (**f**) Taylor diagram comparing the five datasets, summarizing standard deviation, Pearson correlation coefficient, and centered root-mean-square difference. Letters a–e in (**f**) correspond to the datasets in (**a**–**e**), with our dataset as the reference.

The spatial pattern of our natural grassland ANPP dataset was validated against four published datasets. Qualitatively, our estimates align broadly with prior products (Fig. 7a–e), capturing the established biogeographic gradient: high ANPP in the savannas of central Africa and eastern South America, and low ANPP on the Tibetan Plateau.

**Fig. 8:**
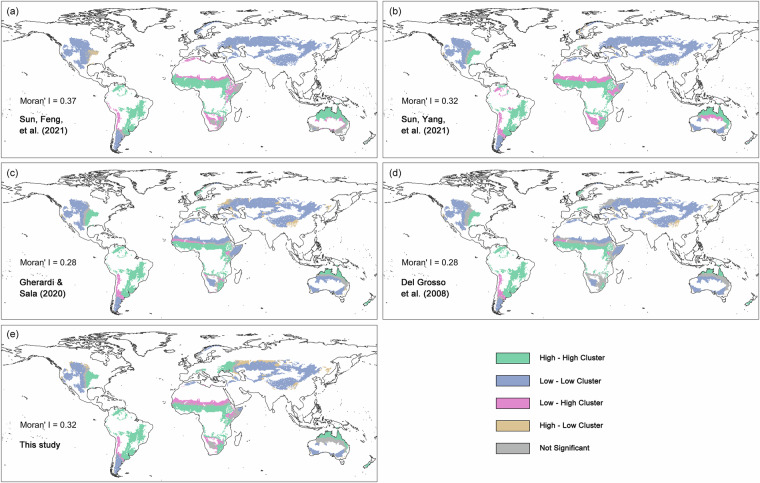
Spatial autocorrelation patterns of global grassland aboveground net primary productivity (ANPP) datasets. Local indicators of spatial association (LISA) cluster maps and global Moran’s I statistics are shown for (**a**–**d**) published datasets and (**e**) our dataset.

Quantitative metrics further support this consistency. First, Kappa statistics (after classifying ANPP into five percentile-based levels) show substantial agreement with existing datasets, with values ranging from 0.65 to 0.74 (all > 0.6). Second, a Taylor diagram (Fig. 7f) demonstrates strong spatial correlations (all > 0.8) between our dataset and each previous study, while its standard deviation falls within the range of the others, confirming realistic spatial variability. Third, global Moran’s I analysis reveals a moderate, reasonable degree of spatial autocorrelation (I = 0.32), comparable to published datasets (I = 0.28–0.37). Local spatial cluster analysis revealed that core areas of high-high and low-low clustering show strong congruence across all datasets (Fig. 8).

Despite overall spatial concordance, our ANPP estimates differ systematically from previous studies: they exceed Del Grosso *et al*.^45^ but are lower than Sun, Feng *et al*.^8^, Sun, Yang *et al*.^44^, and Gherardi & Sala^46^. This divergence arises primarily from underestimation in high-productivity regions (>700 g m^−2^, Fig. 6a), where field measurements are scarce (fewer than 5% of training samples). Limited training data reduced model accuracy in these areas. While our dataset reliably captures spatial patterns and global gradients, users should exercise caution when interpreting absolute ANPP magnitudes in high-productivity ecosystems such as African and South American savannas. Additional field measurements in these underrepresented regions are needed to reduce model uncertainty and improve prediction accuracy.

### Temporal reliability assessment

**Fig. 9:**
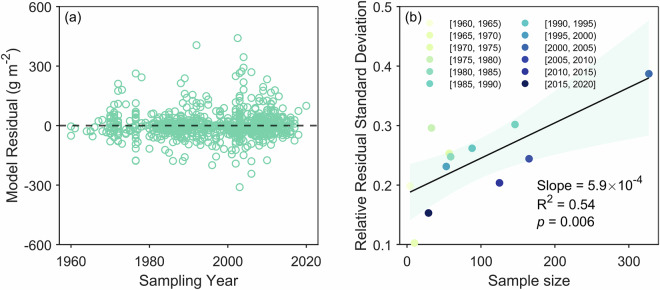
Temporal reliability assessment based on residual analysis. (**a**) Changes in model residuals with sampling years in this study. (**b**) Changes in relative residual standard deviation with sample sizes. The model residual represents the difference between observed and predicted aboveground net primary productivity (ANPP), and the relative residual standard deviation represents the standard deviation of model residuals divided by the mean observed ANPP every five years.

We employed three methods to validate the temporal reliability of our ANPP dataset. First, we examined the relationship between model residuals (differences between observed and predicted ANPP values) and sampling years. Results show that residuals are evenly distributed around the y = 0 line across all sampling years (Fig. 9a). However, unusually large or small residuals occasionally occurred in specific years (e.g., 2003). This can be attributed to the theoretical normal distribution of residuals, where outliers are more likely to occur with larger sample sizes or higher ANPP observations. To further verify this, we partitioned the sampling years into five-year intervals and analyzed the correlation between relative residual standard deviation (the standard deviation of residuals divided by the mean observed ANPP) and sample size within each interval. We revealed that the sample size well explains the residual variation after correcting for observations (Fig. 9b, R^2^ = 0.54, *p* = 0.006). These findings confirm no significant temporal patterns in model residuals, indicating robust model performance without year-specific over- or underestimation.

**Fig. 10:**
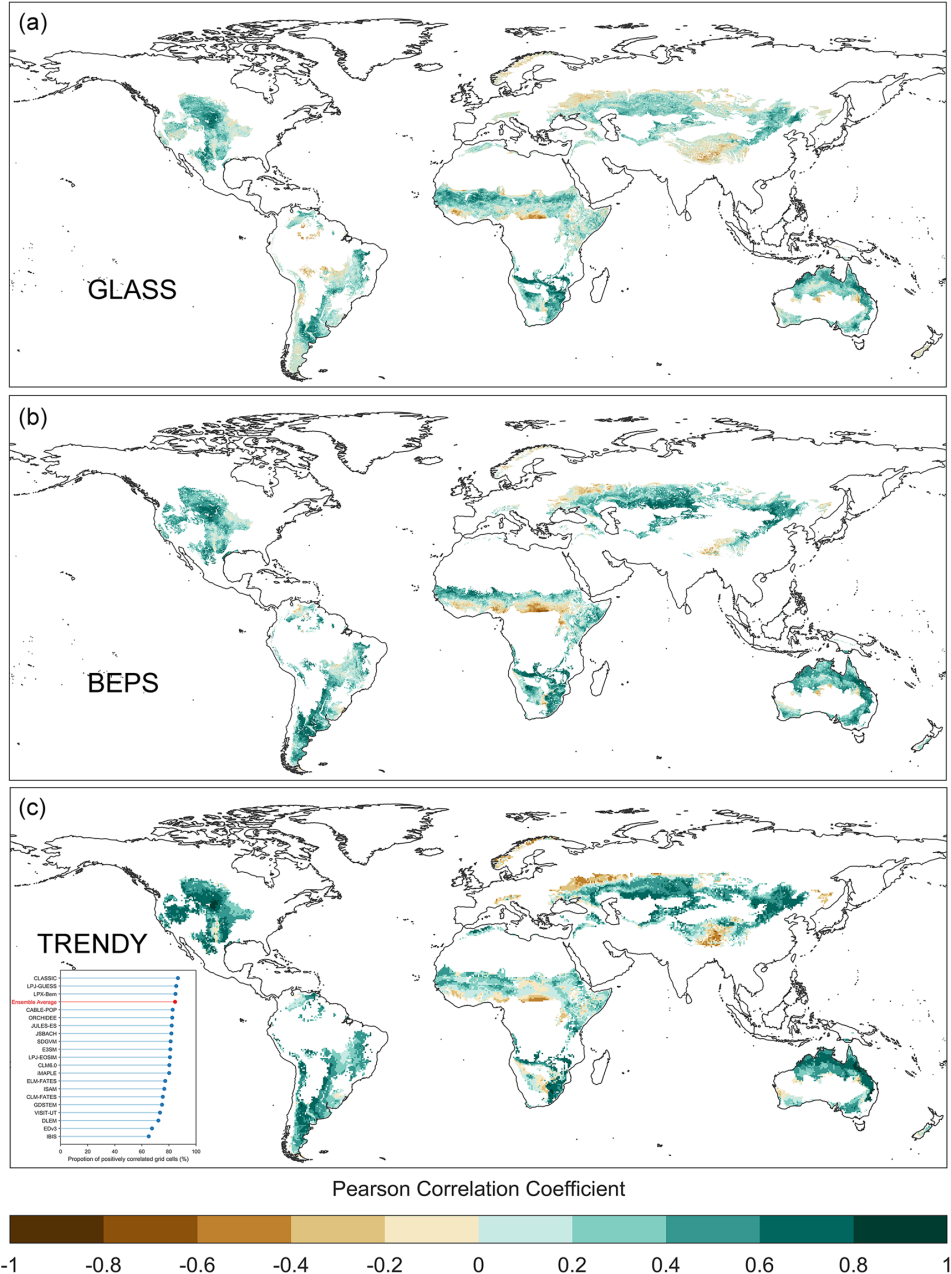
Temporal reliability assessment against satellite-derived net primary productivity (NPP) products and dynamic global vegetation models (DGVMs) simulations. Maps show Pearson correlation coefficients between our aboveground net primary productivity (ANPP) dataset and (**a**) GLASS NPP (1982–2018), (**b**) BEPS NPP (1981–2019), and (**c**) TRENDY ensemble mean NPP (1958–2023). The superimposed plot in (**c**) exhibits the percentage of grassland grids with positive correlation between our ANPP and NPP from each of the 20 individual DGVMs, and the ensemble mean.

We employed two long-term (~40-year) satellite-derived NPP datasets (GLASS and BEPS) to assess whether our ANPP dataset exhibits comparable interannual variability. Grid-level correlations between our ANPP and annual NPP from these products show strong temporal consistency: 78% of grassland grids exhibit positive correlations with GLASS NPP, and 86% with BEPS NPP (Fig. 10a,b). This indicates that our model effectively captures interannual grassland productivity dynamics.

Complementing satellite-based validation, comparison with 20 TRENDY DGVMs provides independent support for the temporal reliability of our dataset. Our ANPP correlates positively with the TRENDY multi-model ensemble mean NPP over 85% of global natural grassland area (Fig. 10c). Among individual DGVMs, 18 of 20 models exhibited positive correlations in >70% of grids (Fig. 10c), with the CLASSIC model achieving the highest agreement (87% positive correlation). This consistency across diverse DGVMs strongly suggests that our dataset robustly captures climate-driven interannual variability in grassland productivity, supporting its use for long-term trend analysis and climate-impact studies.

Nevertheless, persistently negative correlations between NPP and ANPP are observed in the East Sudanian savanna, Tibetan Plateau alpine steppe, and East European forest steppe across multiple datasets. These patterns reflect region-specific mechanisms. In mixed herbaceous-woody systems, woody expansion can raise total NPP^53^ while suppressing herbaceous productivity through competition^54^. On the Tibetan Plateau, warming-enhanced evapotranspiration^20^ and water availability shifts induced by permafrost degradation^55^ promote belowground allocation^56^, increasing total NPP but reducing ANPP. Such discrepancies highlight the need for region-specific interpretation. We recommend local validation in these areas and using observed mismatches to guide further mechanistic research.

## Usage Notes

This study provides a global gridded annual ANPP dataset for natural grasslands. It distinguishes itself from existing NPP datasets by focusing exclusively on aboveground NPP, a rarely isolated metric in global-scale products. Unlike satellite-derived products that reflect post-disturbance conditions, our dataset explicitly models ANPP under undisturbed, natural grassland conditions. This dataset’s temporal resolution (annual) and coverage (extending back to 1958) exceed those of mean-annual studies, e.g., Sun, Feng *et al*.^8^, and shorter-term remote sensing products, e.g., the MOD17 product. Its simplicity—rooted in accessible model inputs and transparent machine learning workflows—enhances reproducibility and broad applicability across ecological and agricultural research.

This dataset supports a range of applications, including but not limited to climate change analysis, carbon allocation investigation, and anthropogenic impact evaluations. Long-term, high-resolution ANPP data enable spatiotemporal assessments of grassland productivity trends, interannual variability, and regional hotspots under climate change. When combined with remote sensing NPP products, the dataset clarifies spatial and temporal patterns in above- vs. below-ground carbon allocation, revealing vegetation adaptation strategies. By contrasting natural ANPP estimates with field-measured or satellite-derived post-disturbance grasslands, researchers can quantify human impacts (e.g., grazing or land use change), assess grassland carrying capacity, and inform sustainable management.

This dataset represents natural grassland ANPP and should not be used to estimate total NPP or ANPP of disturbed grasslands. Please note that this dataset exhibits systematic underestimation in high-productivity regions (ANPP > 700 g m^**–**2^), particularly in the savannas of Africa and South America, due to the scarcity of field observations from these ecosystems in the training data (<5%). Users should therefore exercise caution when conducting quantitative analyses in these regions. We strongly recommend that applications in high-productivity areas be calibrated and validated with local field measurements to correct for potential bias.

## Data Availability

The data supporting this study and the ANPP dataset generated in this study are publicly available at 10.5281/zenodo.18171957.

## Code availability

The *R* code supporting this study is available at 10.5281/zenodo.18171957.
